# Facts Relative to Pemphigus

**Published:** 1791

**Authors:** R. B. Blagden

**Affiliations:** Surgeon at Petworth in Sussex.


XI. *Facts relative to Pemphigus. Communicated in a Letter to Dr.* Simmons *by Mr.* R. B. Blagden, *Surgeon at Petworth in Suſſex.*

I WAS, in January, 1790, deſired to ſee a girl, two years and a half old, who had been obſerved to droop the preceding day. The child looked heavy, and was feveriſh. A neutral mixture with tartariſed antimonial wine was directed for her. The next day the child was more feveriſh, and had ſlight delirium: an enema was then injected, and a bliſter put between tween the ſhoulders. Late in the evening of that day puſtules were obſerved on the waiſt, and before the morning on the other parts of the body, and on the extremities, but chiefly on the forehead and ſcalp. Within three days the puſtules became complete little bladders, being grown turgid with a yellowiſh fluid.

The general ſize of the veſicles was that of an almond, but the ſize of thoſe on the forehead and waiſt approached nearly to that of a nutmeg.

The larger ones were pierced, the ſmaller ones were ſuffered to burſt of themſelves, and not a veſicle filled again.

The unguentum ceræ was made uſe of as dreſſing. The veſicles on the extremities and on the head were healed in about ten days. It was very evident that the child ſuffered extremely from the ſoreneſs of thoſe on the waiſt, which were not completely healed in leſs than two months. The veſtiges of five of the largeſt of theſe, and of two on the forehead, will ever remain.

All the hair, which grew underneath the veſicles on the ſcalp, came off with the plaſters.

Many very minute veſicles were obſerved in the mouth, and the child ſhowed a repugnance to ſwallow. As the fever and delirium went off on the appearance of the eruption, the medicine was laid aſide. No freſh puſtule appeared after the fourth day.

Five days after the appearance of the eruption on this child, an infant, fourteen weeks old, belonging to the ſame parents, grew feveriſh, and, within the next three days, had an eruption exactly reſembling the above, except in the ſize of the veſicles, which, in this caſe, were no larger than peaſe; they burſt, and the parts were well in about a week.

This child was not ill enough to require any medicine internally.

And now may I be permitted to draw the following concluſions:——

That the diſeaſe is contagious;

That new veſicles do not, in every caſe, ariſe after the end of the fourth day;

That the fluid they contain does not, even in every caſe of pemphigus ſimplex, appear to be of a bland nature; and

That, in ſome inſtances, no apparent abſorption of it takes place?

*Petworth,*

May 5, 1791.

XII. *Ac-*

